# Current understanding of electroautotrophy and its relevance in astrobiology‐related research

**DOI:** 10.1002/mlf2.70032

**Published:** 2025-10-15

**Authors:** Quansheng Wang, Maggie C. Y. Lau Vetter

**Affiliations:** ^1^ Department of Extraterrestrial Ocean Systems Institute of Deep‐sea Science and Engineering, Chinese Academy of Sciences Sanya China; ^2^ University of Chinese Academy of Sciences Beijing China

**Keywords:** astrobiology, bioelectrochemical systems, electroactive microorganisms, electroautotrophy, extracellular electron transfer

## Abstract

Electroautotrophy—the use of extracellular electrons as the primary energy source for autotrophic metabolism—remains understudied compared to photoautotrophy and chemoautotrophy. Its occurrence in deep‐earth and deep‐sea environments suggests profound implications for astrobiology, yet electroautotrophic microorganisms remain poorly explored. This review synthesizes the discovery of electroautotrophs and current knowledge from laboratory and field studies, including insights from the deep biosphere. We evaluate their ecological roles on Earth and discuss their potential significance in possible life‐supporting ecosystems elsewhere and in life‐detection strategies. Finally, we propose six key research priorities to advance the study of electroautotrophy in astrobiological contexts.

## INTRODUCTION

Energy is critical for sustaining all life. The most commonly known energy‐gaining modes of autotrophs are phototrophy and chemotrophy. Phototrophic microorganisms capture light energy through photosynthetic reaction centers, whereas chemotrophic microorganisms obtain energy by oxidizing inorganic substances (such as NH_4_^+^, NO_2_^−^, S^0^, H_2_S, H_2_, and Fe^2+^). Photoautotrophs and chemoautotrophs play important roles in Earth's ecosystems as primary producers, providing heterotrophs with organic carbon sources and driving biogeochemical cycles. In recent years, studies have reported that some microorganisms can directly obtain and use electrons for growth and proliferation; these microorganisms are commonly referred to as electroactive microorganisms (EAMs)^1^. EAMs reduce extracellular redox‐active molecules via outer‐surface electron transport chains, interacting with cells and minerals^2^. These EAMs, mainly bacteria and archaea, can acquire electrons directly from conductive solids, a process known as electrotrophy. Electrotrophy can be further classified into electroheterotrophy or electroautotrophy, depending on the carbon source used. The classification of these prokaryotes is summarized in Table 1.

**Table 1:** Classification of prokaryotes according to the source of energy, carbon source and electron donor.

Energy source	Electron donor	Carbon source	Type	Mechanisms	Example
Sunlight Photo‐	Organic ‐Organo‐	Organic compounds ‐Heterotroph	Photoorganoheterotroph	Electromagnetic energy is converted to chemical energy; both electrons and carbon are obtained from the oxidation of organic molecules.	Purple sulfur bacterium (strain M9)^4^
		CO_2_ ‐Autotroph	Photoorganoautotroph	Electromagnetic energy is converted to chemical energy, and the electrons obtained from the oxidation of organic molecules are used for CO_2_ fixation.	Purple Bacteria^5^
	Inorganic ‐Litho‐	Organic compounds ‐Heterotroph	Photolithoheterotroph	Electromagnetic energy is converted to chemical energy, electrons are obtained from oxidation of inorganic molecules, but carbon is obtained from oxidation of organic molecules.	Purple sulfur bacterium *Chromatiaceae* ^6^
		CO_2_ ‐Autotroph	Photolithoautotroph	Electromagnetic energy is converted to chemical energy, and the electrons obtained from the oxidation of inorganic molecules are used for CO_2_ fixation.	Green sulfur bacterium *Chlorobaculum tepidum* ^7^
Chemical compounds Chemo‐	Organic ‐Organo‐	Organic compounds ‐Heterotroph	Chemoorganoheterotroph	Energy, electrons, and carbon are obtained from the oxidation of organic molecules.	*Thermosipho ferrireducens* ^8^
		CO_2_ ‐Autotroph	Chemoorganoautotroph	The energy and the electrons obtained from the oxidation of organic molecules are used for CO_2_ fixation.	Anaerobic methane‐oxidizing archaea^9^
	Inorganic ‐Litho‐	Organic compounds ‐Heterotroph	Chemolithoheterotroph	Energy and electrons are obtained from the oxidation of inorganic molecules, but organic molecules are used as a carbon source.	*Arcobacter peruensis* ^10^
		CO_2_ ‐Autotroph	Chemolithoautotroph	The energy and the electrons obtained from the oxidation of inorganic molecules are used for CO_2_ fixation.	*Ramlibacter lithotrophicus* ^11^
Cathode Electro‐	Extracellular electrons	Organic compounds ‐Heterotroph	Electroheterotroph	Energy and electrons are obtained from outside the cells, but organic molecules are used as a carbon source.	*Clostridium pasteurianum* ^12^
		CO_2_ ‐Autotroph	Electroautotroph	The energy and the electrons obtained from outside the cells are used for CO_2_ fixation.	*Candidatus* Tenderia electrophaga^13^

Adopted and modified from Rago and Harnisch^3^.

Electroautotrophic microorganisms are capable of using CO_2_ as the sole carbon source and extracellular electrons as the primary energy donor^13^, ^14^, ^15^, ^16^, ^17^. A growing number of electroautotrophic microorganisms are being discovered. For example, *Acidithiobacillus ferrooxidans*, which is a typical chemoautotroph, has been shown to exhibit electroautotrophic behavior under electrochemical cultivation conditions^18^. Current knowledge about electroautotrophs is mainly based on culture‐dependent studies and, to a much lesser extent, in situ observations. The recent report of electroautotrophy in natural environmental settings on Earth^19^ has led us to propose that it is possible for electroautotrophy to occur on Mars and icy moons. With the increasing volume of relevant studies, we consider that it is timely to provide a concise summary organized in the following five topics: (1) definitions of electrotrophy and electroautotrophy; (2) the discovery of electroautotrophy; (3) pathways of extracellular electron transfer (EET) and extracellular electron uptake (EEU); (4) devices for studying EAMs and their applications in natural environments; and (5) the significance of electroautotrophic microorganisms to the Earth's ecosystem and implications for astrobiology.

## WHAT ARE ELECTROTROPHY AND ELECTROAUTOTROPHY?

As previously introduced, microorganisms that directly use extracellular electrons as their energy donor are called electrotrophic microorganisms or electrotrophs. The electron donors include microbial cells of different species, iron and other metal ions, and solid electrodes. For example, in bioelectrochemical systems (BESs), electrotrophic microorganisms can directly obtain electrons from the cathode that acts as the electron donor, and transfer them to the terminal electron acceptor either intracellularly or extracellularly^20^. It is important to note that it is not counted as electrotrophy when the electron donors (such as H_2_ or formate) have entered the cells before being oxidized^20^.

Electroautotrophy is one kind of electrotrophy, through which microorganisms synthesize organics from CO_2_ using energy obtained directly from outside of the cells^18^. In BES, electroautotrophic microorganisms use the cathode as the sole electron donor, coupling with NO_3_^−^, SO_4_^2−^ or O_2_ as the terminal electron acceptor, and CO_2_ as the sole carbon source for biomass generation^13^, ^14^, ^15^, ^21^, ^22^, ^23^, ^24^, ^25^.

## THE DISCOVERY OF ELECTROAUTOTROPHY

### Emergence of microbial fuel cell (MFC), microbial electrolysis cell (MEC), and microbial electrosynthesis (MES)

**Figure 1:**
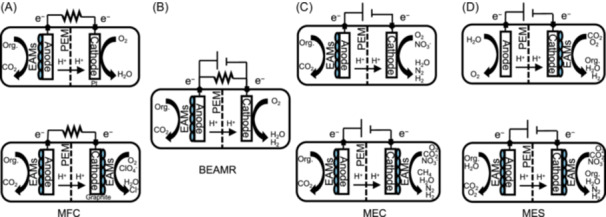
Development of bioelectrochemical systems has led to the discovery of electroautotrophic microorganisms. (A) Electricity‐generating microbial fuel cells (MFCs) began with the use of electroactive microorganisms (EAMs) at the anode (i.e., *bio*anode) pairing with precious metal catalysts as the cathode (upper); and later the expensive nonbiological cathode was replaced with EAMs on inexpensive materials (i.e., *bio*cathode) (lower). PEM, proton exchange membrane. (B) Bioelectrochemically assisted microbial reactors (BEAMRs) then emerged, which inspired the development of microbial electrolysis cells (MECs). (C) With electropotential poised, MEC would employ *bio*anode (upper) or *bio*anode and *bio*cathode (lower) for generating diverse chemical products. (D) For producing organic products, microbial electrosynthesis (MES) systems were developed, with the use of *bio*cathode (upper) or *bio*anode and *bio*cathode (lower). Electroautotrophs are enriched on the *bio*cathodes in MEC and MES. Org. represents organics.

The discovery of electroautotrophy can be traced back to the incorporation of biological materials in electrochemistry systems (Figure 1). In short, firstly it was MFC with *bio*anode and non‐*bio*cathode, then followed by MFC with *bio*anode and *bio*cathode; and at about the same time, MEC with *bio*anode and *bio*cathode emerged. Finally, the research on cells attaching to the *bio*cathode in MES led to the discovery of electroautotrophic microorganisms. While in all of these electrochemical cells, reductive reactions mediated by a biocatalyst (microorganisms or enzymes) occur at the *bio*cathodes, the focus is on different aspects of the reductive reaction. In MFC, *bio*cathodes were sought as an effective alternative cathodic material that enhances electricity production from the anode, and little or no attention was paid to the reductive reaction. In MEC, the reductive reactions on *bio*cathodes were mainly studied to improve the production of fuel gases H_2_ and CH_4_ ^26^, ^27^, ^28^. In MES, emphasis was first paid on the microbial synthesis of biomolecules, and later the desire for a deeper understanding of the bioelectrochemistry of the reductive reaction (i.e., electron‐uptake by the microorganisms on the *bio*cathodes) gave rise to the proposal of electroautotrophic microorganisms^29^.

The experimental setup developed by Michael Potter in 1911 resulted in the observation of spontaneous current flow between the anodic and cathodic electrodes that were immersed, respectively, in bacterial cultures (as the *bio*anode) and sterile media (as the abiotic cathode)^30^. Based on this setup, it was later developed into the first MFC system^31^. Then, Bennetto and Allen designed the MFC system that is commonly used nowadays^32^. Kim and co‐workers^33^ modified MFC, which allowed electroactive bacteria to use electrodes as electron acceptors. The first enrichment of microbial communities that generate electricity, that is, dumping electrons to the anode, via MFC, was achieved in 2004^34^. The electroactive bacterial enrichments have the ability to produce valuable compounds from organic and inorganic wastes^35^. Since then, MFC has drawn increasing attention as it demonstrates promising prospects in sustainable energy production and wastewater treatment^36^.

It was gradually discovered that MFC does not have to be anaerobic, and sustainability of MFC can be improved by using O_2_ as the terminal electron acceptor^37^, ^38^, ^39^ (Figure 1A, upper). But the reduction of O_2_ to H_2_O at the cathode is hindered by kinetics, so a catalyst, usually platinum metal, is needed to speed up the reaction. However, platinum is a rare and very expensive metal, which increases the economic cost of MFC^40^. A viable alternative in MFC is to use microorganisms as biocatalysts at both the anode and the cathode^40^, ^41^ (Figure 1A, lower). Several *bio*cathodic processes that may occur in MFC have been proposed^35^. The versatility of *bio*cathodes allows the use of O_2_ as well as pollutants (e.g., perchlorate) as possible electron acceptors, enabling the removal of pollutants and bioremediation while generating electricity^42^, ^43^.

Bacteria could be used to make H_2_ in an electrolytic process based on a modified MFC. The initial design of microbial H_2_ production in the reactors was reported in 2005^44^. When O_2_ was present at the MFC cathode, current could be produced, but without O_2_, current generation was not spontaneous. This limitation was overcome by applying an external potential (>0.25 V) between the cathode and *bio*anode, enabling H_2_ production at the cathode. As these systems were specifically designed for microbial H_2_ generation rather than electricity production, they were not exactly MFC; therefore, they were termed bioelectrochemically assisted microbial reactors (BEAMRs) or biocatalyzed electrolysis systems^45^ (Figure 1B). This H_2_‐producing process, known as electrohydrogenesis or microbial electrolysis, uses bacteria as exoelectrogens that release electrons to the anode instead of producing H_2_ directly^46^. These reactors represent the precursor to MEC, which emphasizes the electrically driven H_2_ evolution reaction, distinguishing the process from conventional fermentation^46^, ^47^.

Inspired by BEAMR, the development of an H_2_‐producing *bio*cathode from a mixed culture of EAMs became the first to demonstrate the principle of MEC^48^. More specifically, a microbial biofilm that oxidizes acetate and H_2_ would first be developed on the anode, and then, by switching the direction of current flow, the previously designated anodic electrode would become the cathode in MEC (Figure 1C, upper). MEC has the function of producing chemical products (H_2_ or CH_4_); however, for chemical products such as H_2_ generation at the cathode, an additional potential between the anode and the cathode must be applied to overcome thermodynamic limitations^46^, ^48^. Driven by an external potential, MEC keeps supplying the cathode with electrons through oxidation of organics at the anode^43^. When using an active culture as a biocatalyst on the cathode (Figure 1C, lower), the microorganisms (single species or mixed cultures) must meet some basic requirements: it should be able to overcome thermodynamic limitations and generate H_2_ by externally applied voltage; it is able to use the electrode surface as an electron donor and can reduce H^+^ in the medium to H_2_, CH_4_ and other desired chemical products^49^, ^50^. MEC is considered to be more environmentally friendly than MFC in terms of its carbon footprint, as CO_2_ is emitted in MFC, whereas CO_2_ is being fixed at the MEC *bio*cathode^51^.

By using MEC reactors, CO_2_ can be converted into CH_4_ and even other organics at the *bio*cathode. A seminal work^52^ published in 2010 demonstrated that *Sporomusa ovata* had the ability to use electrons derived from a graphite electrode as the sole electron donor for reducing CO_2_ to acetate and small amounts of 2‐oxobutyrate. They proposed the term “microbial electrosynthesis” for the reduction of CO_2_ to multi‐carbon compounds with electrons donated to the microbial cells from an electrode^52^ (Figure 1D, upper). Shortly after, MES was defined more broadly as “the microbially‐catalyzed synthesis of chemical compounds in an electrochemical cell”, which includes the electrochemical reduction or oxidation of other organic feedstocks in addition to the electrochemical reduction of CO_2_ ^53^ (Figure 1D, lower). Generally speaking, in MES, microorganisms use electrons from the cathode to drive reductive reactions. Broadly defined, bioelectrosynthesis includes the harvesting of H_2_, CH_4_, and CH_2_O_2_, as well as electro‐fermentation and fixation of CO_2_ with the creation of multi‐carbon compounds. More specifically, MES refers strictly to systems where CO_2_ fixation is the primary reaction^54^. One should note that electrotrophic microorganisms on the cathode are the core of MES for catalyzing CO_2_ conversion to a wide array of valuable products^55^. These mixed microbial consortia and pure cultures of specialized microorganisms, natural or genetically modified, can act as biocatalysts to reduce CO_2_ by uptaking electrons either directly from the cathode or through extracellular mediators such as H_2_, CH_2_O_2_, NH_3_, Fe^2+^, or even more complex molecules such as self‐produced flavins^56^, ^57^.

It is worth mentioning that the use or labeling of electrodes as cathode and anode, or *bio*cathode and *bio*anode, may change according to different experimental purposes. In MFC, *bio*anode and cathode are generally used, but *bio*anode and *bio*cathode are also used. The function of the *bio*cathode is to accelerate the reaction of catalyzing the reduction of O_2_ to H_2_O; otherwise, a cathode made of inert and expensive metals is required for catalysis. In MEC, *bio*anode and *bio*cathode are generally used, but *bio*anode and cathode are also used. Suppose the purpose of the MEC is to generate H_2;_ in that case, to accelerate the catalytic reaction at the cathodic electrode, one chooses between some precious metals or alloys and low‐cost *bio*cathodes. If the purpose of the MEC is to produce CH_4_, a *bio*cathode is required. In MES, anodes and *bio*cathodes are generally used. A hydrolysis reaction occurs on the anode, and H^+^ reacts at the *bio*cathode, where they combine with CO_2_ to synthesize organic compounds. A *bio*anode can also be used, where organic matter is converted into CO_2_ and H^+^ under the action of microorganisms, and the H^+^ reacts at the *bio*cathode and combines with CO_2_ to synthesize organic compounds^56^.

To summarize, as the understanding of the behavior of microorganisms growing on cathodes in BESs grew, electroautotrophy and electroautotrophic organisms were noticed and began to receive attention.

### Evidence for the existence of electroautotrophic microorganisms and electroautotrophy revealed by isolates and enrichment cultures

As the application of microorganisms on the cathode is an interesting and feasible alternative to precious metals, the use of *bio*cathodes has gained popularity. Electrocatalytic reduction of O_2_ to H_2_O occurs at the *bio*cathode, and it is kinetically limited by the availability of protons because 4 protons are required per reduction of one O_2_ molecule^38^, ^58^. When studying *bio*cathodes at low pH, such *bio*cathodes have the advantage of overcoming the kinetic limitation because more H^+^ ions are available when compared to circumneutral and alkaline conditions. Hence, acidophilic microorganisms that can utilize O_2_ as the terminal electron acceptor at electrodes are preferred. *A. ferrooxidans* is a good candidate and has remarkably shed light on our understanding of electroautotrophs. It is microaerobic and chemoautotrophic, which uses CO_2_ as the carbon source and derives energy from the oxidation of Fe^2+^, S^0^, or reduced sulfur compounds at low pH using O_2_ as the terminal electron acceptor^59^. It was found that *A. ferrooxidans* grows on the solid cathodic electrode as the sole energy source, with an applied voltage of 0 V (vs. Saturated Calomel Electrode, SCE), without any redox species among the electrolytes, and such a *bio*cathode exhibits electrocatalytic properties of O_2_ reduction^60^.

Since the chemoautotrophic *A. ferrooxidans* has shown impressive electrotrophic capabilities in MFC and MEC, attempts were made to further explore its additional electrotrophic capabilities. *A. ferrooxidans* was the subject of the study that introduced for the first time the concept of “electrolithoautotrophy”, which was hypothesized as possibly the third type of energy‐gaining process^18^. The study provided empirical evidence that the chemoautotrophic Fe^2+^‐oxidizing *A. ferrooxidans* exhibits electroautotrophic growth on the solid cathodic electrode with electrons as the primary source of energy.

Based on our extensive review of the literature about isolating and culturing electroautotrophic microorganisms, to the best of our knowledge, electroautotrophic behavior was actually first reported in the model exoelectrogen genus *Geobacter* ^61^, ^62^. The authors observed that exoelectrogens have the ability to extract electrons from electrodes and use them to reduce uranium. This ability makes exoelectrogens useful in the treatment of uranium mine wastewater, as they can aid in removing and recovering uranium. Additionally, exoelectrogens might have a role in treating chlorinated compounds, nitrates, and other toxic metals. Nevertheless, the research at the time emphasized pollution control; no attention was paid to electroautotrophy.

Acetogens and methanogens are found to be electroautotrophic, and both are strictly anaerobic microorganisms. Acetogenic *Sporomusa ovata* was first described by MES to reduce CO_2_ by using electrons accepted from the cathode^52^. Then, several other microorganisms, including other *Sporomusa* sp. as well as *Clostridium* sp. and *Moorella* sp., and even archaeal species such as *Methanobacterium* sp. and *Methanococcus* sp., were proposed for MES use^63^. Acetogens and methanogens are popular in MES studies because of their growth characteristics. Acetogens are facultative autotrophs capable of heterotrophic growth through oxidation of a diverse range of organic substrates, such as hexoses, pentoses, and alcohols, or autotrophic growth through oxidation of inorganic substrates like H_2_ or CO, coupled with CO_2_ reduction. Methanogens mostly utilize CO_2_ as the carbon source and H_2_ as the electron donor through hydrogenotrophic methanogenesis. In addition, certain methanogens are capable of utilizing CO for methanogenesis^64^.

*Desulfosporosinus orientis* and *Desulfovibrio piger* are the first examples of sulfate‐reducing microorganisms (SRMs) that really showed authentic electroautotrophy, when the cultures were poised with the cathode potential of −310 mV (vs. standard hydrogen electrode [SHE]) and CO_2_ was the sole carbon source^65^. *Desulfopila corrodens* IS4 is the first Fe^0^‐corroding SRM that showed direct electron uptake from the solid electrode, with CO_2_ as the carbon substrate^66^. *Desulfobacterium autotrophicum* HRM2 was also reported in BES on their electroautotrophy (−500 mV vs. SHE) along with bioelectrosynthesis of acetate^67^. The majority of SRMs are able to use H_2_ as an electron donor in chemotrophy, so the abiotic H_2_ can mediate the EET in EAMs^68^. Mechanisms of EET are discussed in a later section.

*Thioalkalivibrio nitratireducens* (in the order *Ectothiorhodospiraceae* of the class *Gammaproteobacteria*), a halo‐alkaliphilic facultative denitrifier, which originated from the sediments of soda lakes, exhibited electroautotrophic growth in the absence of oxygen by using nitrate as an electron acceptor, even though it prefers aerobic conditions^69^. Similarly, another denitrifier *Thiobacillus denitrificans* has also demonstrated its capability of electroautotrophic growth through electron uptake from the BES cathode^65^. *Alcaligenes faecalis*, which survives in oxygenated or oxygen‐lacking environments and has versatile carbon and nitrogen metabolisms, such as ammonium oxidation, nitrogen fixation, and denitrification, has the capacity to both generate electricity and perform denitrification through bidirectional EET^70^. Its electroautotrophic process was supported by inward EET (i.e., electron uptake) coupling with nitrate reduction^70^.

*Rhodopseudomonas palustris*, a typical photosynthetic bacterium, can perform direct interspecies electron transfer (DIET) and inward EET for photosynthesis. The electron source supporting autotrophic growth of *R. palustris* in the absence of light was observed to be supplied by *Geobacter metallireducens* ^71^. This electric syntrophic coculture managed to thrive in the dark and oxygen‐depleted environment, a condition that neither of the strains would have been able to grow without one another^71^. Then *R. palustris* was individually evaluated in BES. Under anaerobic conditions, either illuminated or in the dark, *R. palustris* was found to take up from the cathode extracellular electrons that entered the photosynthetic electron transport chain and resulted in the activity of ribulose‐1,5‐bisphosphate carboxylase/oxygenase (RuBisCO) for CO_2_ fixation via the Calvin‐Benson‐Bassham (CBB) cycle, confirming the coupling of inward EET and photosynthesis^72^, ^73^. Notably, the discovery of electroautotrophy in *R. palustris* suggested the potential for anaerobic dark carbon fixation in conventional photosynthetic bacteria^71^, and broadened the ecological niche of electroautotrophic bacteria.

It is believed that not all electroautotrophs are obtained through the domestication of axenic chemoautotrophic isolates. There are a considerable number of examples of successful enrichment of electroautotrophic microorganisms directly from environmental samples. One prominent example is the self‐regenerating and self‐sustaining cathodic biofilm *Bio*cathode‐MCL, named after the three dominant bacterial species *Marinobacter*, *Chromatiaceae*, and *Labrenzia* ^74^, that was enriched from seawater^75^. *Bio*cathode‐MCL grew aerobically on metal electrodes or graphite cathodes, and at relatively high potential (+310 mV vs. SHE), which precluded the possibility that H_2_ generated from electrolyzed water could promote cell growth^76^. It was demonstrated that electrons move across the electrode and biofilm interface, and then enter >5 μm into the biofilm, likely fueling biomass formation from CO_2_ fixation^14^. Follow‐up omics analyses have provided more comprehensive insights into the primary producers, described as “electroautotrophs”^13^, which to date have not been isolated in axenic cultures. The complete genome sequence of the *Labrenzia* sp. strain CP4 suggested that *Bio*cathode‐MCL exhibits electroautotrophy via the pathways of EET^21^. In addition, the metagenome‐assembled genome of another active, putatively novel species “*Candidatus* Tenderia electrophaga” contains all the genes required for CO_2_ fixation via the CBB cycle, including two forms of RuBisCO (IAc and IAq), as well as homologous genes for electron transfer and O_2_ reduction similar to the aforementioned electroautotrophic *A. ferrrooxidans* ^13^. Metagenomics and proteomics confirmed the EET and CO_2_ fixation mechanisms of “*Ca*. T. electrophaga,” while metatranscriptomics similarly elucidated the electroautotrophic mechanism of “*Ca*. T. electrophaga”^22^. Several genes encoding the EET pathway of “*Ca*. T. electrophaga” are more highly expressed at high potentials (+470 mV vs. SHE compared to +310 mV vs. SHE), and the protein encoding Cyc2 is homologous to that of *A. ferrooxidans*, which is known to be involved in electroautotrophy (+400 mV vs. SHE)^18^. These results strongly indicated that “*Ca*. T. electrophaga” is the key electroautotrophic organism in the *Bio*cathode‐MCL community^22^. This “*Ca*. T. electrophaga” is a candidate taxon within *Gammaproteobacteria* that has not yet been axenically cultured despite several attempts. Comparative genomics suggested that the order *Tenderiales* contains a conserved cluster named *uet*ABCDEFGHIJ, encoding the EEU protein, and electroautotrophy may be widespread within *Tenderiales* ^25^.

Electroautotrophs originate not only from mesophilic samples but also from extreme environments. A novel H_2_‐oxidizing bacterium, *Kyrpidia spormannii*, was isolated from a thermoacidophilic electrotrophic community enriched at 60°C from geothermal hot spring sediments^15^. Stable carbon isotopic analysis confirmed the electroautotrophic growth ability of this thermoacidophilic biofilm on the cathode^15^. Furthermore, when grown under electroautotrophic, lithoautotrophic, and heterotrophic conditions, transcriptomic analyses of *K. spormannii* revealed differential expression profiles in the conversion of CO_2_ to polyhydroxybutyrate (PHB) and in energy conservation^15^. Hyperthermophilic microbial enrichments were obtained through incubation under electroautotrophic conditions in the laboratory at 80°C^24^ as well as in situ at 314°C at a hydrothermal vent. It was hypothesized that the electron flow of these microorganisms can be sourced from hydrothermally generated redox potential across the chimney wall^77^.

To summarize, the main indicative aspects of electroautotrophic growth are: (1) in the absence of electron‐donating chemical species, the electrode in the electrochemistry cells acts as the main energy donor; (2) there is a significant electron outflow from the cathode compared to the negative controls, suggestive of electrons being consumed by the EAMs that have shown corresponding increase in their populations; (3) CO_2_ or bicarbonate is the sole carbon source supporting the growth of the EAMs; (4) the magnitude of cathodic current consumption varies with different electron acceptors, and diverse overall biochemical reactions may occur at the cathode; and (5) last but not the least, under the condition of electrochemical enrichment culture in the laboratory, the cathodic H_2_ production can be excluded by thermodynamic calculation to rule out the possibility of microbial chemoautotrophic growth resulting from H_2_ diffusion into the cells and subsequent oxidation.

### Expanding the diversity of electroautotrophs

**Figure 2:**
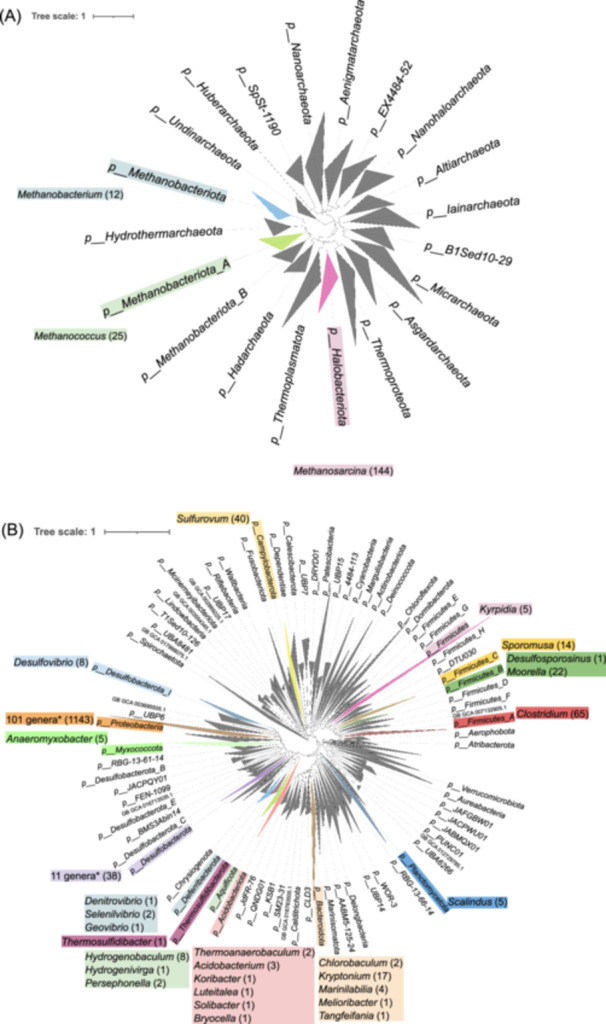
Diversity of microorganisms with the genetic potential for electroautotrophy or bioengineered electroautotrophy. The base trees for Archaea (A) and Bacteria (B) were downloaded from Genome Taxonomy Database (GTDB) release R207. According to GTDB's documentation, the archaeal and bacterial reference trees were inferred from the concatenation of 53 and 120 genes, respectively. Phyla containing putative electroautotrophs, as suggested in Figure 5 in Abel et al.^87^, are highlighted. The genome identifiers and detailed taxonomic classifications are provided in Table S1.

In addition to the few examples of methanogens, acetogens, nitrate reducers, sulfate reducers, and metal reducers highlighted above, isolates exhibiting electroautotrophic ability in MES are listed in Table 2. While the listed organisms represent diverse chemotrophic energy metabolisms, their taxonomic representation appears to be limited. Recently, through data mining, taxonomically diverse genomes with the genetic potential for electroautotrophy or for bioengineered electroautotrophy have been proposed^87^. A total of 181 archaeal genomes and 1396 bacterial genomes were identified based on the identification of marker genes for the CBB cycle, reductive tricarboxylic acid (rTCA) cycle, respiratory nitrate reduction factor (Rnf) respiratory enzymes, denitrification, cytochrome *c* oxidase, and electron conduits. To get a sense of the diversity of electroautotrophic candidates within the context of the current knowledge of the tree of life, we looked up the lineages of these genomes and visualized them in the archaeal and bacterial trees shared by Christopher Rinke of Genome Taxonomy Database (GTDB) (https://itol.embl.de/shared/mealworm). The trees are collapsed at the phylum level, highlighting the diverse phyla and genera represented by the genomes identified in Abel and co‐workers^87^ and in Table 2 (Figure 2, Table S1).

**Table 2:** Summary of major electroautotrophic microorganisms related to MES.

Microorganism	Domain/phylum^a^	Electron acceptor	Cathode as electron donor	Cathode potential (V)	Reference
**Methanogens**					
*Methanobacterium palustre*	Archaea/*Methanobacteriota*	CO_2_	Carbon cloth	−0.70 vs. Ag/AgCl	^78^
*Methanosarcina barkeri*	Archaea/*Halobacteriota*	CO_2_	Platinum	−0.65 vs. Ag/AgCl	^79^
*Methanobacterium* sp. IM1	Archaea/*Methanobacteriota*	CO_2_	Graphite rod	−0.40 vs. SHE	^80^
*Methanococcus maripaludis*	Archaea/*Methanobacteriota*	CO_2_	Graphite rod	−0.70 vs. SHE	^81^
*Methanococcus vannielii*	Archaea/*Methanobacteriota*	CO_2_	Graphite rod	−0.70 vs. SHE	^81^
*Methanosarcina petrolearia*	Archaea/*Halobacteriota*	CO_2_	Graphite rod	−0.70 vs. SHE	^81^
*Methanoculleus submarinus*	Archaea/*Halobacteriota*	CO_2_	Graphite rod	−0.70 vs. SHE	^81^
*Methanobacterium congolense*	Archaea/*Methanobacteriota*	CO_2_	Graphite rod	−0.70 vs. SHE	^81^
**Acetogens**					
*Sporomusa ovata*	Bacteria/*Firmicutes*	CO_2_	Graphite stick	−0.40 vs. SHE	^52^
*Sporomusa silvacetica*	Bacteria/*Firmicutes*	CO_2_	Graphite stick	−0.40 vs. SHE	^52^
*Sporomusa sphaeroides*	Bacteria/*Firmicutes*	CO_2_	Graphite stick	−0.40 vs. SHE	^52^
*Sporumosa malonica*	Bacteria/*Firmicutes*	CO_2_	Graphite stick	−0.69 vs. SHE	^52^
*Clostridium ljungdahlii*	Bacteria/*Firmicutes*	CO_2_	Graphite stick	−0.40 vs. SHE	^52^
*Clostridium aceticum*	Bacteria/*Firmicutes*	CO_2_	Graphite stick	−0.40 vs. SHE	^52^
*Moorella thermoacetica*	Bacteria/*Firmicutes*	CO_2_	Graphite stick	−0.40 vs. SHE	^52^
***Geobacter***					
*Geobacter metallireducens*	Bacteria/*Desulfobacterota*	NO_3_^–^	Graphite	−0.50 vs. Ag/AgCl	^62^
*Geobacter sulfurreducens*	Bacteria/*Desulfobacterota*	C_4_H_2_O_4_^2^^–^	Graphite	−0.50 vs. Ag/AgCl	^62^
**Other bacteria**					
*Mariprofundus ferrooxydans* PV‐1	Bacteria/*Proteobacteria*	CO_2_	Graphite	−0.076 vs. SHE	^82^
*Rhodopseudomonas palustris*	Bacteria/*Proteobacteria*	CO_2_	Carbon brush	−0.40 vs. SHE	^73^
*Kyrpidia spormannii*	Bacteria/*Firmicutes*	CO_2_	Graphite felt	−0.53 vs. SHE	^15^
*Pseudomonas alcaliphila* MBR	Bacteria/*Proteobacteria*	NO_3_^–^	Carbon felt	−0.30 vs. Ag/AgCl	^83^
*Alcaligenes faecalis*	Bacteria/*Proteobacteria*	NO_3_^–^	Carbon cloth	−0.50 vs. SHE	^70^
*Thioalkalivibrio nitratireducens*	Bacteria/*Proteobacteria*	NO_3_^–^	Carbon cloth	−0.25 vs. Ag/AgCl	^69^
*Thiobacillus denitrificans*	Bacteria/*Proteobacteria*	NO_3_^–^	Graphite rod	−0.50 vs. Ag/AgCl	^65^
*Desulfovibrio ferrophilus* IS5	Bacteria/*Desulfobacterota*	SO_4_^2–^	ITO‐glass	−0.40 vs. SHE	^84^
*Desulfopila corrodens* IS4	Bacteria/*Desulfobacterota*	SO_4_^2–^	Graphite rod	−0.40 vs. SHE	^66^
*Desulfosporosinus orientis* *Desulfovibrio piger*	Bacteria/*Firmicutes*	SO_4_^2–^ SO_4_^2–^	Graphite rod Graphite rod	−0.50 vs. Ag/AgCl −0.50 vs. Ag/AgCl	^65^
	Bacteria/*Desulfobacterota*				
*Desulfobacterium autotrophicum* HRM2	Bacteria/*Desulfobacterota*	SO_4_^2–^	Graphite	−0.50 vs. SHE	^67^
*Acidithiobacillus ferrooxidans*	Bacteria/*Proteobacteria*	O_2_	FTO‐glass	+0.40 vs. SHE	^18^
*Rhodopseudomonas palustris* TIE‐1	Bacteria/*Proteobacteria*	O_2_	Graphite	+0.10 vs. SHE	^85^
*Ralstonia eutropha* H16	Bacteria/*Proteobacteria*	O_2_	NiMoZn	+0.85 vs. Ag/AgCl	^76^
*Nitrosomonas europaea*	Bacteria/*Proteobacteria*	O_2_	Copper	+0.12 vs. Ag/AgCl	^86^

FTO, fluorine doped tin oxide coated glass; ITO, indium tin oxide coated glass; MES, microbial electrosynthesis; SHE, standard hydrogen electrode.

^a^ Taxonomic classification follows Genome Taxonomy Database (GTDB) release R207.

*Protoebacteria* remains to be the leading phylum hosting the majority of potential electroautotrophs. This analysis added 21 genera and 8 phyla (namely *Myxococcota*, *Deferribacterota*, *Thermosulfidibacterota*, *Aquificota*, *Acidobacteriota*, *Bacteroidota*, *Plactomycetota,* and *Campylobacterota*) that have not been previously characterized as electroautotrophs. It is believed that as experiments are performed to validate electroautotrophy by these microorganisms, more holistic views on the biodiversity of genuine electroautotrophs will be revealed. With that, we can gain further insights into the inventory of molecular machineries, and mechanisms and regulations of potentially different types of microbial EEU. Such data will enable us, for example, to find out the relative occurrence between obligate versus facultative electroautotrophs, to investigate whether some EEU systems and pathways are specific to certain phylogenetic groups and/or certain habitats, and if so, to seek what might be the determining factors that explain such specificities, and perhaps to infer the evolution of EEU.

## PATHWAYS OF EET

**Figure 3:**
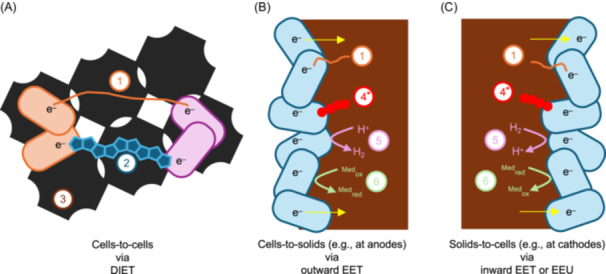
Summary of different extracellular electron transfer (EET) pathways. While it is common to classify EET based on the nature and function of the mediators, here the emphasis is on the means of electron transport. (A) Electrons are transported between cells via direct interspecies electron transfer (DIET) through (1) direct contact via conductive pili and/or nanowires, (2) conductive particles smaller than the cells (e.g., magnetite), and (3) conductive particles larger than the cells (e.g., activated carbon). (B) Electrons are exported from cells to the solid surface via outward EET through (1), (4) direct contact via redox‐active proteins, (5) diffusive soluble electron shuttles, and (6) redox mediators or enzymes. (C) Electrons are taken up by cells from the solid surface via inward EET or extracellular electron uptake (EEU) through (1), (4)–(6). Asterisks denote that not the exact same mechanism is employed by outward EET and inward EET when redox‐active proteins are involved.

Since microbial cell membranes are not permeable to insoluble minerals or electroconductive material, microorganisms have strategically evolved to exchange electrons with extracellular insoluble minerals by a process known as EET^88^. Although EET describes the bidirectional electron transfer in and out of the microorganisms, as microbial electroactivity of electron export from microorganisms to anodes, or termed as “outward EET” in Karbelkar et al.^89^, was first known and studied more extensively, and thus consequently, the pathways of “outward EET” are better known than other EET processes^90^. According to our current understanding, more specifically, EET can be classified into three types: outward EET, inward EET, and DIET (Figure 3).

### Outward EET

For outward EET, microorganisms export their electrons through (1) direct contact via conductive pili and/or nanowires; (2) direct contact via redox‐active proteins such as *c*‐type cytochromes; (3) diffusive soluble electron shuttles such as endogenous mediators (flavins, phenazines, and quinones) and natural substances (humic acid), as well as artificial substances (neutral red, anthraquinone‐2, 6‐disulfonate); and (4) redox mediators or enzymes (H_2_ and CH_2_O_2_, or enzyme like hydrogenases, as well as formate hydrogenases)^2^, ^88^, ^91^.

### Inward EET (or EEU)

The microbial capacity to use electrons from insoluble electron donors, such as redox‐active minerals, poised electrodes, or even other microbial cells, is called EEU^92^. In brief, the currently known EEU mechanisms include the following: (1) direct contact via redox‐active proteins (e.g., c‐type cytochromes or nanowires/pili); (2) small molecules (e.g., H_2_, CH_2_O_2_ and endogenous or artificial redox mediator like flavins, quinones, anthocyanin, and thionine) acting as electron mediators, meaning that they are not oxidized within the cells; and (3) electron mediators generated by extracellular enzymes (e.g., hydrogenases or formate dehydrogenases) that are attached on cathodes^56^, ^89^, ^93^ (Figure 3). We consider that EEU can be further classified into direct extracellular electron uptake (DEEU) and indirect extracellular electron uptake (IEEU) (Figure S1). DEEU primarily performs via cytochromes and nanowires. If there are multiple layers of cells present at the electrodes, electrons will be conducted through the intermediate cells to the outermost cells^94^. As for IEEU, electron mediators are essential.

As of now, the five models of EEU‐driven microbial autotrophy are (1) Cyc2‐based model in acidophilic and neutrophilic chemoautotrophs; (2) MtoAB‐based model in microaerophilic and anaerobic chemoautotrophs; (3) Multiheme cytochrome *c* (MHC)‐based model in sulfate reducing and methanogenic chemoautotrophs; (4) PioAB‐based model; and (5) Forkhead box protein (FoxEYZ)‐based model in anoxygenic photoautotrophs^92^. It is worth noting that inward EET is currently less well‐known than outward EET. The complete process by which the electrons are taken up by microorganisms from cathodes is not a simple reverse of the outward EET pathways^89^, and it is still a topic of debate.

Inward EET pathway in *Shewanella oneidensis* MR‐1 cannot be simply explained by a reversal of its outward EET pathway^95^. According to the results of gene knockout experiments that eliminate EEU via the outward EET pathway, two previously unknown mechanisms were proposed for the transfer of cathodic electrons from the Mtr EET complex to the ubiquinone pool and onto terminal cytochrome oxidases. A similar distinction exists in *Geobacter sulfurreducens*: while outward EET relies on the inner membrane cytochrome ImcH or CbcL, which transports electrons to the periplasm via a transport system, where they are received by periplasmic cytochromes that function as electron transmitters, and the final step involves the transfer of electrons to electrodes by the outer membrane *c*‐type cytochrome omcZ and other alternative outer membrane cytochromes (OMCs), inward EET follows a fundamentally different route. Although the periplasmic *c*‐type cytochrome (PccH) is the only cytochrome reported as being critical for the inward EET process^96^, ^97^, outer membrane *c*‐type cytochromes (*omcS*, *omcC*, and *omcB*) are expressed at lower levels during inward EET than during outward EET, indicating that these *c*‐type cytochromes may not be functional in inward EET^98^, ^99^. The complete mechanism of inward EET in *Geobacter* remains unresolved.

*c*‐type cytochromes are employed by iron‐oxidizing bacterium *A. ferrooxidans* in the “down‐hill (exergonic)” pathway for generating the proton motive force (PMF), and meanwhile, 1/15 of the electrons are channeled to the PMF‐dependent up‐hill electron transfer for the conversion of NAD^+^ to NADH in the CBB cycle for CO_2_ fixation^18^. A recent multi‐omics study showed that *A. ferrooxidans* simultaneously employs multiple ways to facilitate EEU, including *c*‐type cytochromes, transmembrane proteins (pilin and porin) with direct electron transfer capacity, and extracellular polysaccharide substances^100^.

For organisms that can perform both outward and inward EET, the mechanisms are still being studied. While MtrAB system is better known as an outward EET pathway, its bidirectional function has been hinted by genomic analysis of Fe(III)‐reducing and Fe(II)‐oxidizing bacteria *Shewanella* spp.^101^. Yet, for SRM *Alcaligenes faecalis* that is known to perform outward EET and generate electricity at a poised potential (+300 mV vs. SHE), and also is able to carry out electroautotrophic denitrification at −500 mV vs. SHE, the proteomic profiles of its outward EET biofilm were found to be significantly different from those of its inward EET biofilm, with pili and outer membrane proteins probably being responsible for outward and inward EET, respectively. Therefore, it can be inferred that the different electron transport conduits of *A. faecalis* can be utilized for bidirectional EET^70^.

Less information on the inward EET of Archaea is available. Cyclic voltammetry revealed the involvement of redox‐active components (cytochrome *b* and *c*) that facilitated electron transfer from the electrode to a *Methanobacterium*‐like archaeon strain IM1 during electromethanogenesis^80^. It has been shown that *Methanosarcina barkeri* is capable of interacting with cathodes through hydrogenase‐mediated and extracellular enzyme‐independent modes, as well as a yet‐to‐be‐described direct interaction mechanism^102^. An uncultured archaeal acetogen “*Candidatus* Serpentinarchaeum aceticum” was found to express highly the genes encoding for a MHC and pilin, hinting at the use of some pathways for direct use of extracellular electrons^103^.

### DIET

Another kind of EET is DIET between microorganisms, a cell‐to‐cell electron transfer between species through a shared connection^104^. The electron donor and acceptor form a mutualistic relationship through DIET, allowing them to jointly accomplish metabolic processes that cannot be achieved by a single microorganism^105^. Since the initial discovery of DIET^106^, it has been increasingly recognized that microorganisms that are able to establish electron transfer with their partners are uniquely advantageous under some environments, and microbial DIET may also have a significant role in biogeochemistry cycling^107^. At present, the only example of DIET‐supported electroautotroph is *R. palustris* which depends on *G. metallireducens* solely for energy supply under laboratory condition^108^. Therefore, we believe that there are more electroautotrophs being able to live on DIET partnership, not only in the laboratory, but also in natural environments. Some electroautotrophs rely on their DIET partners for electron supply, and also, some may acquire extracellular electrons from solid substrates and donate electrons to their DIET partners.

DIET mechanisms include the following: (1) direct contact via conductive pili and/or nanowires; (2) conductive particles smaller than cells (e.g., magnetite); and (3) conductive particles larger than cells (e.g., activated carbon)^1^, ^107^ (Figure 3).

EET between species occurs primarily through the use of redox mediators (H_2_, formate, acetate, sulfur compounds, and quinones), pili or pilus‐like structures^91^. It is also reported that two porin‐cytochrome complexes, PccF and PccG, have a significant role in DIET between *G. metallireducens* and its coculture^1^. However, PccG seems to be more critical than PccF in terms of its growth with *Methanosarcina*. Unlike cocultures with *G. sulfurreducens* and *M. acetivorans*, the presence of electrically conductive pili was not essential for the growth of *G. metalliredufcens* with *M. barkeri* ^109^. By contrast, *S. oneidensis*, another electroactive microbe with plentiful outer‐surface *c*‐type cytochromes, which is commonly regarded as a model microbe equivalent to *Geobacter* species, was not capable of DIET with *Methanosarcina* species. The results showcased the fact that having outer‐surface *c*‐type cytochromes does not always imply the ability for DIET, especially in different species^109^.

Pili play a structural but not a conductive function in supporting *Geobacter* biofilm formation^110^. In contrast, the omcS nanowire plays a conductive but not a structural function in facilitating electron transfer in the biofilm^111^. The omcB nanowire plays both a structural and a conductive function to contribute to electrotrophy^111^, ^112^, ^113^.

## DEVICES FOR STUDYING EAMs AND THEIR USE IN NATURAL ENVIRONMENTS

### Basic designs of BES

Electrochemical technology and electrochemical devices are essential for studying EAMs. Microbial electrochemical techniques are employed to exploit the electrochemical interactions of microorganisms and electrodes^114^. BES is an electrochemical platform that uses microorganisms as electrode catalysts. BES has been applied to understand mechanisms of electroautotrophic growth^18^ and anaerobic syntrophic organisms^108^, as well as in situ chemolithoautotrophic microbial communities^115^.

As introduce above (Figure 1), MFC generates electricity, MEC and MES generate chemicals and organics, respectively; they have become typical representatives of BESs^51^, ^116^. Generally speaking, the anode and cathode of MFC are separated by a membrane (e.g., proton exchange membrane, PEM) or salt bridge, yielding a design known as a two‐chamber system. Organics are used as fuel and are oxidized by microorganisms in the anaerobic anode chamber, and protons move across the PEM and react with O_2_ in the cathode chamber to generate H_2_O^117^. MEC and MES cells are essentially composed of two electrodes (cathode and anode), commonly separated by a membrane like PEM. An oxidation process takes place at the anode (e.g., organics oxidation or H_2_O oxidation), whereas a reduction process occurs at the cathode (e.g., O_2_ reduction or H_2_ evolution). MEC and MES differ in design from MFC in that MFC is a dual‐chamber system, whereas MEC and MES can be either single or dual‐chamber systems. For single chamber systems, the electrodes are surrounded by electrolyte, the fluid around the electrode containing the reactants and products, which is generally an aqueous solution or wastewater (as a feed source)^52^, ^53^, ^54^.

Early studies of EAMs were mainly performed using 2‐electrode BES, and later, 3‐electrode BES became more common because of its greater flexibility and amenability. In microbial 3‐electrode cells (M3Cs)^43^, the working electrode (WE) potential is set using a potentiostat against a reference electrode (RE) (e.g., Ag/AgCl), and is connected to the counter electrode (CE) to complete the circuit. The WE potential can be set to either the optimal anode potential (for cells‐to‐anodes reactions) or the optimal cathodic potential (for cathodes‐to‐cells reactions).

**Figure 4:**
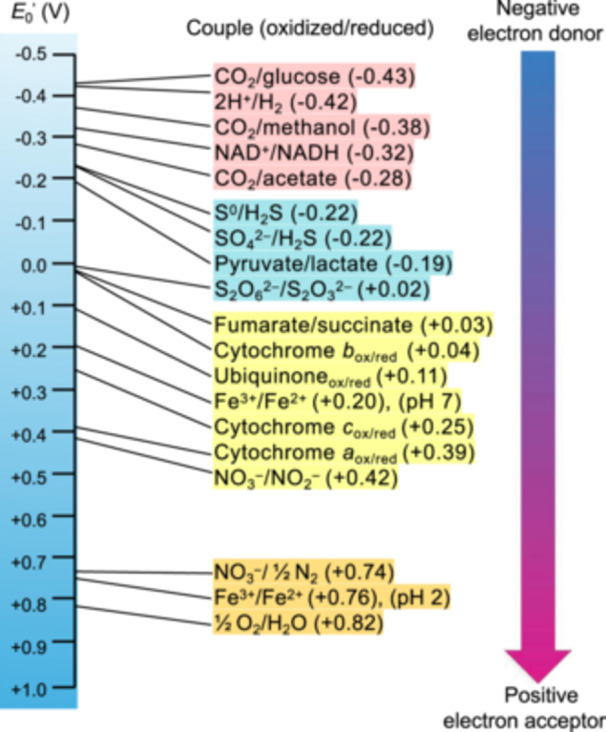
The redox potential tower. Cell potentials given are under standard state conditions with [ions] = 1 M, temperature = 25°C (298.15K), and gas pressure = 1 atm (101.325 kPa). Electrons spontaneously move from donors with more negative reduction potentials (*E*_0_' vs. SHE at pH 7) to acceptors with more positive reduction potentials. Data from Lee et al.^118^ and Roginska et al.^119^.

BES is essentially an electric cell with redox reactions, simulating various metabolic activities. Many of the redox potentials (*E*_0_^'^) of half‐reactions that can be mediated by microorganisms are within the stability limits of H_2_O (Figure 4). According to the Nernst equation, the upper stability limit of water at pH 7 can be estimated through the electrolysis of H_2_O molecules, pO_2_ = 0.21 atm ((1/2) H_2_O ↔ H^+^ + (1/4) O_2_ + e^−^), *E*_0_^'^ = (0.0592/1) log {pO_2_^1/4^ /(10^−7^)^2^} = 0.819 V (vs. SHE). Likewise, the lower stability limit of water at pH 7 can be estimated through the formation of H_2_ gas, pH_2_ = 0.95 atm (H^+^ + e^−^ ↔ (1/2) H_2_), *E*_0_^'^ = −(0.0592/1) log {pH_2_^1/2^ /10^−7^} = −0.414 V (vs. SHE)^118^. Therefore, for studying electrotrophs, electrochemical reactions in a neutral aqueous solution should be given a theoretical voltage within the range between −0.414 V and 0.819 V (vs. SHE). In practice, due to kinetic limitations, a broader range of applied voltages is usually needed to overcome overpotentials and activate electrochemical reactions^120^.

### Detection of electroactive microbial activity in environmental samples and in the field

BES has gained popularity due to its various benefits, including high sensitivity, stability, safety, and portability. It has shown enormous potential and value in many fields, such as environmental bioremediation, energy generation, biosynthesis, biogas upgrading, biosensing, and intelligent devices.

Solid compounds with reduced properties (e.g., FeS and S^0^) found in the natural environment are particularly favorable for enriching microorganisms that are capable of conducting cathodic oxidation^65^. As such, BES enrichment in the laboratory is advantageous for cultivating and studying mineral‐oxidizing microorganisms because the enriched electrotrophic microorganisms rely on the electrodes as the sole electron donor, a surrogate of insoluble minerals, and therefore, can be free of electron‐donating insoluble minerals and/or mineral metabolic products^106^, ^115^.

When poising potentials across the electrodes that were inserted into marine sediment samples to simulate various redox potentials, a current was observed, which increased with more negative redox potential (−50 mV to −400 mV vs. Ag/AgCl)^115^, and eventually a few electrotrophic taxa were obtained. As for other studies, the working electrodes (cathode) were buried in sediment microcosms and maintained at seven different redox potentials ranging from −300 mV to −750 mV vs. Ag/AgCl^121^. After 2 months of incubation, the overall diversity of the community increased with more negative redox potentials, and the abundance of known EET‐capable groups (e.g., *Alteromonadales* and *Desulfuromonadales*) varied with redox potential. EAMs have also been confirmed to be present in the samples obtained from deep terrestrial subsurface^82^, ^122^ and deep marine subsurface^123^, ^124^.

An electrode‐based method for monitoring the on‐site activity of microorganisms during subsurface bioremediation was performed^125^. Despite the fact that the anode and cathode were placed 6 m apart, electrons resulting from the oxidation of acetate were transferred from the *Geobacter* species to the anode, and detectable current was observed for the first time in the field. Currents can, therefore, be used as an effective means to monitor in situ microbial activity in subsurface hypoxic environments^125^. Similarly, the BES principle has been applied to detect and measure in situ microbial activity in hot spring^126^, serpentinization site^127^, ^128^, and terrestrial subsurface^129^. Notably, most of these cited studies used *bio*anodes. More field studies using *bio*cathodes to probe electroautotrophs are strongly encouraged, as the resulting data will provide useful information about electroautotrophic microbial activity under native conditions.

A recent study^19^ conducted in situ electrochemical cultivation using a *bio*cathode and electrical potentials naturally generated at deep‐sea hydrothermal vents. Voltages and currents generated by microbial activity were observed over 12 days of deployment, and a novel bacterium belonging to the genus *Thiomicrorhabdus* dominating the microbial community was enriched exclusively at the *bio*cathode^19^. Metagenomic analysis indicated that this bacterium has thio‐autotrophic growth potential. Thus, this bacterium was proposed as a new species, specifically enriched during geoelectricity generation, as “*Candidatus* Thiomicrorhabdus electrophagus”. This finding suggested that electroautotrophic (or “electrosynthetic” in the paper) microbial populations can naturally occur in deep‐sea hydrothermal environments by using geoelectricity. This result will be a key reference for us in discovering MES‐supported ecosystems in the natural environment.

## SIGNIFICANCE OF ELECTROAUTOTROPHIC MICROORGANISMS TO DIFFERENT ECOSYSTEMS

### Providing a potential solution for sustainability

The relevance of EAMs to biotechnology and bioprospect has received much attention and has been reviewed^57^, ^130^, ^131^, ^132^. Besides, electroautotrophs and their ability to convert CO_2_ into organic compounds via MES are of interest in their economic values^133^ as well as in addressing global issues. Anthropogenic activities leading to high CO_2_ emissions have brought global concern as greenhouse gas CO_2_ contributes to global warming, which is threatening many of the Earth's ecosystems. New ideas for carbon sequestration strategies are in sought^134^, and BES‐aided growth of electroautotrophs is being explored as a potential means to meet the carbon neutralization goals^135^. Yet hurdles, such as upscaling from small bioreactors to industrial size, must be overcome for electroautotrophs to bring significant impact on the global ecosystem. Additionally, electroautotrophs are promising biocatalysts in MES that can meet the demand for renewable energy. Biogas upgrading, the conversion of CO_2_ to CH_4_ via electromethanogenesis, can boost the energy and economic benefits by increasing the CH_4_ content from ~50–70% in untreated biogas to ~80‐98% in treated biogas^136^, ^137^.

### Serving as the primary producers supporting electrotrophic ecosystem(s)

Current has been detected across conductive minerals in deep‐sea hydrothermal vent fields^77^, ^138^, ^139^, which has led to the suggestion that free electrons may be one of the energy sources supporting some deep‐sea microorganisms, in addition to diffusible reducing compounds^18^. It is exciting that the hyperthermophilic microbial communities from deep‐sea hydrothermal vent chimney samples were enriched successfully under electrolithoautotrophic culture conditions in the laboratory^24^ and in natural settings^19^. In addition to chemotrophic and phototrophic ecosystems, deep‐sea hydrothermal environments may be nurturing a new type of ecosystem with electroautotrophs as the primary producers^77^, which we call “an electrotrophic ecosystem”.

Many aspects of electrotrophic ecosystems are still currently unknown. Questions regarding the ecology and evolution of the probable electrotrophic ecosystems remain to be answered: Is there a *bona fide* electrotrophic ecosystem in nature? What are the interactions between members of the chemotrophic and electrotrophic ecosystems? How widespread are electrotrophic ecosystems in the contemporary era? As electron‐donating minerals act like cathodes, they might have supported electroautotrophs to occur in geologically active terrestrial and marine subsurface on a global scale over the geological timescale^92^, ^140^. Then, would autotrophic metabolisms driven by EEU have emerged under early Earth conditions?

Fortunately, some hints of naturally occurring electrotrophic ecosystems slowly emerge. A better understanding can be gained by considering the in situ electroactive microbial activity and electron consumption reported from diverse environments of the deep biosphere, for example, hot spring^26^, serpentinization site^127^, ^128^, deep‐sea hydrothermal vents^19^, and terrestrial subsurface^129^. These reports of microbial electroactivity may have only scratched a tiny bit of the deep biosphere that hosts a vast reservoir of microbial biomass^141^. The discovery of a novel electroautotroph “*Ca*. T. electrophagus” via in situ enrichment at an artificial deep‐sea hydrothermal vent^19^ provides compelling support that an electrotrophic ecosystem fueled by geoelectricity can actually occur in nature. Although this organism has the genetic capacity to acquire energy from reduced sulfur species (e.g. H_2_S) via the SOX pathway, it is believed that at the study site where H_2_S is highly depleted and geoelectricity is available; “*Ca*. T. electrophagus” uses electric energy via EEU to support CO_2_ fixation via the CBB cycle and other cellular functions.

Furthermore, an in‐depth analysis of the genome, representing an uncultured archaeon “*Candidatus* S. aceticum” belonging to the order *Methanocellales*, surprisingly reported the absence of the key functional genes for methanogenesis^103^. Instead, the presence of MHC and pilin, and their exceptional gene expression, strongly suggested that “*Ca*. S. aceticum” fuels acetogenesis through EEU, possibly from highly reduced minerals such as ferroan brucite. Finding “*Ca*. S. aceticum”‐like taxa in both terrestrial and marine serpentine‐hosted fluids provided further evidence that organisms lacking light‐ and chemical‐dependent energy metabolisms and possessing EEU ability can drive an electrotrophic ecosystem in serpentine settings that are common on Earth and other planetary bodies.

### Existence of electrotrophic ecosystem(s) elsewhere?

Similarly, it is projected that a vast volume of continental deep biospheres could exist on other rocky planets, providing the largest, longest‐lived, and most stable environment^142^, making the subsurface habitable zone far greater than that on the surface^143^. Likewise, when it comes to looking for life beyond Earth in our solar system, three of the best candidates (Europa, Titan, and Enceladus) are ice‐covered moons with subsurface oceans of liquid water^144^. The surface of Mars and icy moons is less likely to be habitable due to the extreme temperatures and strong radiation stress^145^, ^146^. In contrast, their subsurface environments are more likely to meet life requirements, and with long‐term nutrient and energy supplies, and a wide range of energy sources (e.g., thermal energy, chemical energy, and electric energy), theoretically suitable for stimulating the formation of various organic compounds^147^, ^148^. With in situ electroactive microbial activity being detected in the Earth's subsurface as aforementioned, electroactive and electroautotrophic microorganisms might also occur on Mars and icy moons.

**Figure 5:**
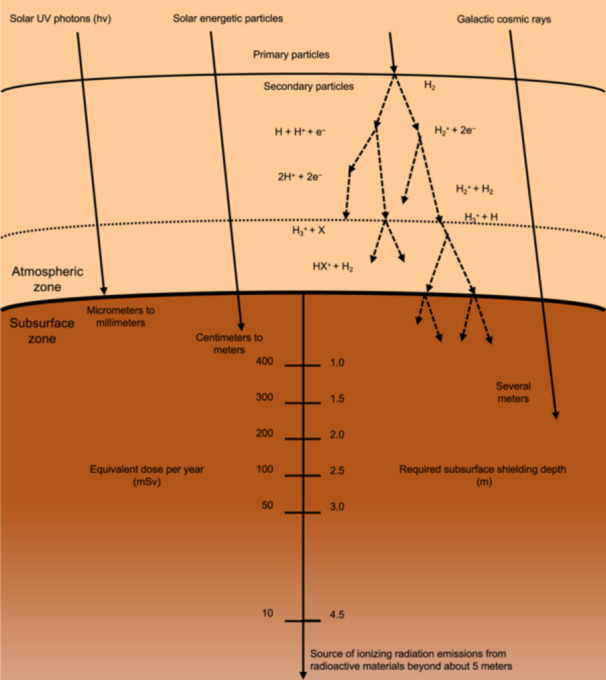
Different kinds of radiation on Mars and ionization processes. Penetration depth of radiation in the shallow subsurface varies. Radiation would result in ionization, thus the production of secondary and tertiary particles. X is a neutral molecule in a nebula, such as CO, H_2_O, and N_2_. According to the World Nuclear Association, the typical background radiation experienced by everyone is a whole‐body radiation dose on average of 2.4 mSv/year, but it varies largely depending on location. For example, the natural background levels in several places in Iran, India, and Europe are 50 mSv/year. Data from Fornaro et al.^150^, Röstel et al.^151^, and Feng^149^.

Free electrons, potentially supporting electrotrophic ecosystems, may exist in Mars's shallow subsurface. At the near‐surface, solar ultraviolet photons, solar energetic particles, and galactic cosmic rays are the main ionization sources; upon absorbing energy, the charge‐neutral atmosphere would be able to form free electrons as electrons are stripped away from gas molecules^149^. Compared to the near‐surface of Mars, at greater depths on Mars, for example, 4–5 m below the land surface, subsurface radionuclides can serve as the primary source of ionization for free electrons formation^150^ (Figure 5). According to the simulations of a sulfate‐concrete‐like geological model of Mars and calculations based on the amount of radiation attenuation^150^, ^151^, one can estimate the penetration depths and doses of different kinds of radiation to suggest the potential depth at which underground life may exist. By considering these simulation results together with the annual radiation dose acceptable by terrestrial organisms, we hypothesize that, despite the thin Martian atmosphere, certain Martian strata located at least 3 m below the land surface may possess sufficiently low levels of radiation and possess free electrons with adequately low energy, making them plausible options for sustaining life.

A geoelectric field might be present in the hydrothermal systems in Enceladus and Europa. The observation of nanosized (<10 nm in radius) silica particles by Cosmic Dust Analyzer^152^, ^153^ in the dust particles indicated fluid–rock interaction within the porous core of Enceladus as an ongoing process, which constrained the pH, salinity, and water temperature at the bottom ocean^154^. For the nanosized silica to precipitate out of the hydrothermal fluid upon mixing with the cooler seawater, the fluid temperature must be at least 90°C (at pH 10.5 and salinity of ~1.5% NaCl) if the fluid temperature remains constant^154^. Should there be pH changes upon mixing, which is very likely, the minimum temperature required to form silica nanoparticles becomes 50°C^155^. Hydrothermal activity is likely to be present in Europa. Plumes were investigated for their contribution to the chaotic regions on the surface^156^. The exact structure of the ocean‐rock interface where mineral deposition occurs when hotter hydrothermal fluids meet with cooler sea water is not known because bathymetry data for Enceladus and Europa are lacking. Nevertheless, it is conceivable that nanoporous mineral structures with structural alignment that allows selective ion transport^157^ exist, resembling those of the Earth's vent chimneys, where geoelectricity established between the reducing hydrothermal fluid and the oxic sea water supports electroautotrophic microorganisms^19^.

**Figure 6:**
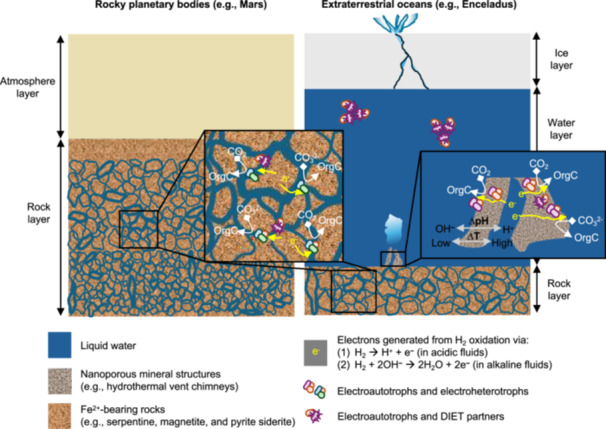
Schematic diagram of electroautotrophs supported in extraterrestrial environments. Water‐rock reactions result in local acidic or alkaline H_2_‐rich fluids, which create an electropotential across the mineral surface. Electrons then flow through the porous structures upon the demand of electroactive microorganisms. Electroautotrophs, depending on geoelectricity or DIET partnership, may occur in rocky planetary bodies and extraterrestrial oceans where other life requirements are also met. OrgC represents organic carbons, ∆ represents gradients in pH and temperature (T).

As alterations of subsurface deposits (e.g., serpentinization) appear to be common in extraterrestrial environments, for example, on Mars^145^, asteroids^158^, and Enceladus^155^, we hypothesize that geoelectricity may occur to support electrotrophic activities, and by inference, such an energy source may support electroautotrophs and their DIET partners to occur (Figure 6). We are aware that these hypotheses, based solely on energy availability, may appear to be inadequate because, for life to thrive, it takes more than energy inputs. If electroautotrophy could take place on Mars and icy moons, could they adapt and survive the extreme conditions? Some of the major electroautotrophic microorganisms listed in Table 2 (e.g., methanogens, SRM, and *A. ferrooxidans*) are prominent examples of microbial life that could live under extraterrestrial‐like conditions, in spite of the fact that their adaptation to and survival under such conditions were illustrated when they grew chemotrophically. While these chemoautotrophs capable of electroautotrophy (i.e., facultative electroautotrophs) can cope with extraterrestrial‐like conditions, further investigations on how electroautotrophically grown microorganisms adapt to and survive in the extreme conditions similar to those on Mars and icy moons would be highly relevant to astrobiological research, and will strengthen our hypotheses.

### Serving as life analogs in astrobiological studies

“Are we alone in this universe?” is a long‐standing question that has driven many scientific and engineering projects to research and search for possible life beyond Earth. Investigations of analog organisms and their environments on Earth can enrich our knowledge in the broad spectrum of survival and adaptation mechanisms, and can inspire instrumental development for life detection.

Earth's extreme acidophilic bacteria serve as key analogs for potential extraterrestrial life because they are able to adapt to and thrive under relevant extreme conditions. Notably, as reviewed by Slowik et al.^159^, *A. ferrooxidans* represents a particularly relevant model organism for the sulfur‐rich and low pH environment in Venus's lower cloud layer. Venus' surface is too harsh to support life, but the habitability of its clouds has remained a focus^160^. The chemical and physical conditions in Venus' lower cloud layers are favorable for *A. ferrooxidans*, such as the presence of sulfur compounds (and the implied high acidity), CO_2_, and H_2_O, and moderate temperatures (0‐60°C) and pressures (0.4–2 atm)^161^, ^162^.

At higher altitudes, if microbial cells exist, they would be frozen but not necessarily killed^163^. A review indicated that, apart from abiotic materials (e.g., SO^2^, S^0^, and FeCl_3_), biological materials also showed reasonable spectral overlap with the spectral signatures observed from Venus, in particular, those containing an Fe‐S cofactor^164^. Iro protein (with an Fe‐S cofactor), which is believed to play a role in iron respiration in *A. ferrooxidans* ^165^, shows significant overlap with Venus' albedo at 300–500 nm^164^. As *A. ferrooxidans* has been shown to metabolize through electroautotrophy, if it survives in the clouds of Venus, electroautotrophy could be one of its energy‐gaining modes.

The active Poás Volcano in Costa Rica, with high temperature fumaroles (up to 980°C) and a pH in −1 to 1.5 super acidic lakes, has been considered a good geochemical analog for acidic environments that are rich in sulfur and sulfates on Mars^166^. Amplicon sequencing and shotgun metagenomic analyses showed that the observed microbial diversity of the volcano is highly dominated (>90%) by a single species in the genus *Acidiphilium*, which likely derives its energy from the oxidation of reduced sulfur^166^, ^167^. *Acidiphilium* sp. was enriched along with *A. ferrooxidans* and other acidophiles on the *bio*cathodes derived from acid‐mine drainage samples^168^. Although further evidence to illustrate direct electron uptake by *Acidiphilium* is pending, it is reasonable to believe that *Acidiphilium*, like other acidophiles, are preferential *bio*cathodic materials that can consume electrons from the electrode and mediate the kinetically‐limiting O_2_ reduction. Additionally, a recent study demonstrated that *A. ferrooxidans* grew chemoautotrophically by using Fe^2+^ that was solubilized from vivianite (proposed, but not confirmed on Mars) and siderite (already confirmed)^169^. Given its known ability to extract electrons from a solid surface, it is likely that *A. ferrooxidans* had directly extracted electrons from these Fe^2+^‐bearing minerals in their experiments. Furthermore, modeling of Europan ocean chemistry suggested the ocean water to be as acidic as pH 2.6 due to high fluxes of oxidants^170^, an environment that is favorable for *A. ferrooxidans*. The above said suggested that electroautotrophic acidophilic bacteria can serve as an invaluable analog for possible life in relict acid‐sulfate hydrothermal systems and iron‐bearing geological formations on Mars, as well as in some extraterrestrial oceans.

Additionally, methanogens, acetogens, and SRM (such as those listed in Table 2) have been the key microorganisms in many astrobiological studies. For example, methanogens survive and produce CH_4_ under Mars‐^171^ and Enceladus‐like^172^ conditions. Methanogenesis and acetogenesis are ancient metabolisms in the Earth's history^173^, ^174^. SRM of considerable population sizes are predicted to be sustained by radiolytic sources on Mars^175^ and the Europan ocean^176^.

With our hypothesis that electroautotrophy may be feasible beyond Earth and the above examples of facultative electroautotrophs having the potential to survive in extraterrestrial‐like environments, we argue that (1) electroautotrophic microorganisms are previously unrecognized terrestrial analogs for investigating possible life elsewhere; (2) bioelectrochemical studies to deepen the understanding of EET will provide new insights into energy conservation strategies in ancient metabolisms; and (3) even if electroautotrophy might not be the default mode of nutrition, electroautotrophic characteristics of (obligate or facultative) electroautotrophs would allow us to probe for such electrical signals as indicative of possible biological signals. Therefore, bioelectrochemical sensors for measuring biological activities should be sought among future life‐detection payload instruments, for their low power demand, capacity for miniaturization, simple packing, and diverse functionality. As a matter of fact, the idea of using geoelectric signals to detect extraterrestrial life has been explored ^177^ because ionic (ex)changes due to metabolic activity could result in detectable changes in electrical conductivity. Thus, dynamic changes in electrical conductivity and in situ current flow could potentially be evidence for a sign of life, provided that abiotic reasons are ruled out. Nonetheless, direct measurements of changes in electrical signals could be affected by many factors, and it would be difficult to distinguish between and/or to tease apart abiotic and biotic contributors. In comparison, to probe for electrotrophic characteristics of electrotrophs, one way is to poise electropotentials and to measure active electron consumption (i.e., elevated current compared to sterile controls). The electropotential applied would affect what active electrotrophic cells, electroactive biomolecules, and/or chemicals could extract electrons from the electrode. To increase our confidence in interpreting current measurements due to biological activities on Mars or elsewhere, we must enhance our understanding of the metabolisms, behaviors, and habitats of electroactive, and more specifically, electroautotrophic microorganisms. It is worth noting that the current signal is often low in on‐site detection; advancement in electrochemical techniques will enable us to capture the weak current signal^178^.

It is worth pointing out that, apart from supporting electrotrophic ecosystem(s) on Earth and possibly beyond, geoelectricity has been reported to be involved in the abiotic formation of organics. Li and co‐workers demonstrated that geoelectrochemical process could account for degradation of amino acids and the resultant formation of monoamines, hydroxy acids and n‐ω‐amino acids in carbonaceous chondrites parent bodies and asteroids^158^, ^179^. A recent perspective article also suggested possible roles that geoelectricity may play at each of the transition steps from prebiotic to the emergence of the first life^180^. These studies also highlighted the significance of electrochemistry in answering questions related to the “origin(s) of life”.

## CONCLUDING REMARKS

Electroautotrophy, although less studied than photo‐ and chemo‐autotrophy, emerges as a metabolic strategy exhibited by diverse microorganisms and detected in different Earth's ecosystems, including the deep biosphere, and potentially beyond. Through this review about electroautotrophy and electroautotrophs, we hope to encourage more research to be carried out to explore their potential role as the primary producers in natural settings, which potentially support an electrotrophic ecosystem that may exist on Earth and/or other extraterrestrial environments. We also hope to see advancement in BES‐inspired instrumentation within the context of astrobiology and space exploration. Research priorities to be considered for the future include, but are not limited to, the following:

- *To elucidate and illustrate the fundamentals of electroautotrophy*. This allows us to harness the basic understanding of its emergence, metabolic efficiency, detailed mechanisms, its relation to phototrophy and chemotrophy, and therefore, to estimate and predict its occurrence in time and space.
- *To identify and characterize more electroautotrophic individuals as a model analog*. This allows us to broaden choices for more focused experiments, and therefore, to gain knowledge about biological activities and adaptation strategies under the constraints specific to, for instance, locales on contemporary or ancient Mars, and icy moons.
- *To expand and confirm the type of habitats that support an electrotrophic ecosystem*. This allows us to realize how ubiquitous such ecosystems may be, and therefore, to evaluate how likely electrotrophic ecosystems may have existed or persist in our universe.
- *To develop technologies for validating biotic EEU*. This allows us to differentiate between electron loss or consumption via abiotic versus biotic processes.
- *To design and fabricate (payload) instruments for detecting electrical signals and specific metabolites of electroautotrophs*. This allows us to gain new observations that may suggest new phenomena and viewpoints regarding biological activities beyond Earth. Parallels should be set up to assess abiotic factors that may interfere with biological signals, and thus cause false positives.
- *To contribute to a sustainable life‐supporting system for astronauts in future manned space missions*. This allows us to use electroautotrophs to recycle the anthropogenic CO_2_ and synthesize nutritional supplements for astronauts, and that may enhance extraterrestrial habitability, and thus, extend the length of their stay and travel in the space environment.

Cross‐disciplinary efforts—spanning bioelectrochemistry, microbiology, geochemistry, ecology, and space engineering—are essential to unravel electroautotrophy's role in Earth's ecosystems, both ancient and modern, and its astrobiological significance in potential extraterrestrial biospheres. We anticipate that advancing the study of electroautotrophs will illuminate new frontiers in understanding habitability on Earth and beyond.

## Supporting information


**Figure S1 Schematic representation of direct EEU (DEEU) and indirect EEU (IEEU) from the cathode by electroactive bacteria.** The graphic focuses on the extracellular electron uptake process rather than the complete pathway of inward EET.

**Table S1 List of putative electroautotrophs highlighted in the Tree of Life in** Figure 2. Genome identifiers and taxonomic classifications are provided.
