# Supplementary material for: Current understanding of electroautotrophy and its relevance in astrobiology‐related research
Source: mLife. 2025 Oct 15;4(5):473–93. doi: 10.1002/mlf2.70032 (PMC12575090; doi:10.1002/mlf2.70032)
Supplement: Supplementary file 1 — Figure S1 Schematic representation of direct EEU (DEEU) and indirect EEU (IEEU) from the cathode by electroactive bacteria. The graphic focuses on the extracellular electron uptake process rather than the complete pathway of inward EET. [file MLF2-4-473-s002.docx]

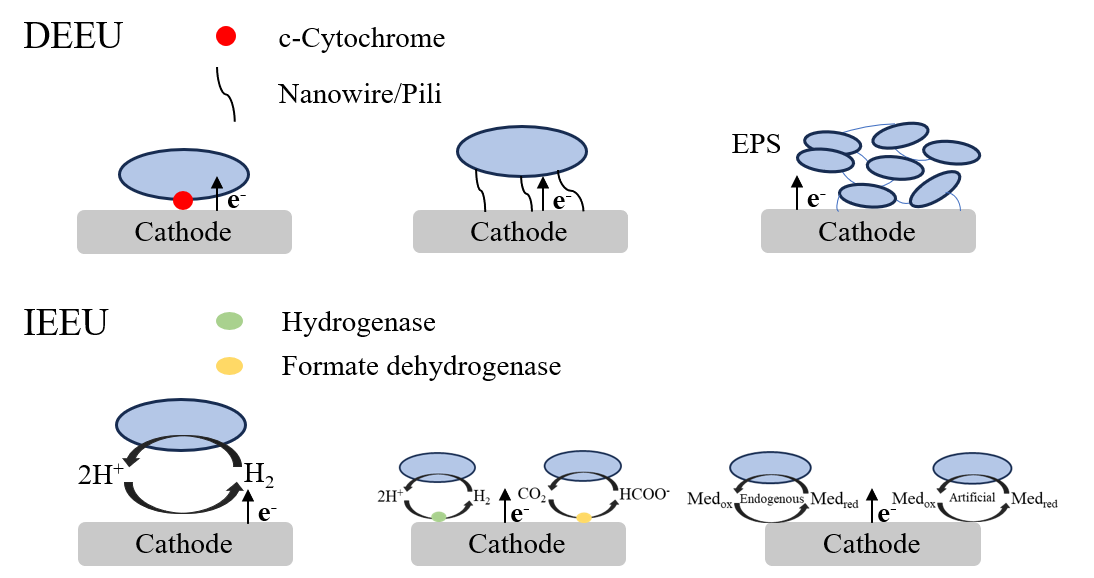


**Figure S1. Schematic representation of direct EEU (DEEU) and indirect EEU (IEEU) from the cathode by electroactive bacteria.** The graphic focuses on the extracellular electron uptake process rather than the complete pathway of inward EET.
